# Alpha‐synuclein mRNA isoform formation and translation affected by polymorphism in the human *SNCA* 3ʹUTR

**DOI:** 10.1002/mgg3.407

**Published:** 2018-05-06

**Authors:** Elizabeth S. Barrie, Sung‐Ha Lee, John T. Frater, Maria Kataki, Douglas W. Scharre, Wolfgang Sadee

**Affiliations:** ^1^ Institute for Genomic Medicine Nationwide Children's Hospital Columbus Ohio; ^2^ Center for Pharmacogenomics Department of Cancer Biology and Genetics College of Medicine The Ohio State University Columbus Ohio; ^3^ Division of Cognitive Neurology Department of Neurology The Ohio State University Wexner Medical Center Columbus Ohio

**Keywords:** 3ʹUTR regulation, allelic expression, alpha‐synuclein, genetic variation, Parkinson's disease, SNCA

## Abstract

**Background:**

Multiple variants in *SNCA*, encoding alpha‐synuclein, a main component of Lewy bodies, are implicated in Parkinson's disease.

**Methods:**

We searched for *cis*‐acting *SNCA* variants using allelic mRNA ratios in human brain tissues. In a *SNCA* 3′UTR (2,520 bp) luciferase reporter gene assay, translation in SH‐SY5Y cells in the presence of the rs17016074* G/A* alleles was measured. To assess clinical impact, we queried neurocognitive genome‐wide association studies.

**Results:**

Allelic ratios deviated up to twofold, measured at a marker SNP in the middle of a long 3′ untranslated region (3′UTR), but not at a marker at its start, suggesting regulation of 3′UTR processing. 3′UTR SNP rs17016074 *G/A*, minor allele frequency (MAF) <1% in Caucasians, 13% in Africans, strongly associates with large allelic mRNA expression imbalance (AEI), resulting in reduced expression of long 3′UTR isoforms. A second 3′UTR SNP (rs356165) associates with moderate AEI and enhances SNCA mRNA expression. The rs17016074 *A* allele reduces overall 3′UTR expression in luciferase reporter gene assays but supports more efficient translation, resolving previous contradictory results. We failed to detect significant genome‐wide associations for rs17016074, possibly a result of low MAF in Caucasians or its absence from most genotyping panels. In the “Genome Wide Association Study of Yoruba in Nigeria,” rs356165 was associated with reduced memory performance.

**Conclusions:**

Here, we identify two *cis*‐acting regulatory variants affecting SNCA mRNA expression, measured by allelic ratios in the 3′UTR. The rs17016074 minor *A* allele is associated with higher expression of luciferase protein activity. Resolving the genetic influence of *SNCA* polymorphisms requires study of the interactions between multiple regulatory variants with distinct frequencies among populations.

## 1 INTRODUCTION

Aggregation of alpha‐synuclein, encoded by *SNCA* (OMIM#163890), also referred to as NACP (nonamyloid component of plaques), is a main component of Lewy bodies, the hallmark of Parkinson's disease (PD) and other Lewy body dementias, and is also implicated in cases of Alzheimer's disease. In PD, numerous significant GWAS signals distribute over the entire gene locus, with strong signals located in the *SNCA* 3′UTR, a region affecting mRNA localization, protein abundance, and protein localization (Berkovits & Mayr, 2015; Sandberg, Neilson, Sarma, Sharp, & Burge, 2008). This holds particular importance for SNCA as different isoforms and expression levels are predicted to affect SNCA aggregation (Bungeroth et al., 2014; Manda, Yedlapudi, Korukonda, Bojja, & Kalivendi, 2014). Moreover, the 3′UTR harbors several miR sites, targeted by multiple miRs including miR7 and miR34b,c, shown to affect alpha‐synuclein expression and related phenotypes (Junn et al., 2009; Kabaria, Choi, Chaudhuri, Mouradian, & Junn, 2015; McMillan et al., 2017; Tatura et al., 2016). Association studies alone have failed to definitively identify all key functional variant(s), because of large haplotype blocks across the gene harboring multiple candidate variants, and significant linkage disequilibrium (LD) in the 3′UTR.

Minor allele frequencies of *SNCA* SNPs vary greatly across racial groups. For example, the frequency of the 3′UTR *G* allele of rs356165, NM_000345.3(SNCA):c.*893C>T, in Europeans and Africans is 36% and 66%, respectively (http://gnomad.broadinstitute.org). Across most populations, the *G* allele is considered the major allele, while it is the minor allele in Europeans. A majority of genetic association studies on cognition and neurodegenerative disorders are restricted by race, with the large meta‐analyses on PDGENE.org focused on Caucasians (Lill et al., 2012; Nalls et al., 2014). Detailed studies on the incidence of PD in Sub‐Saharan Africa are lacking; one group reports that 0.6% of participants had a diagnosis of PD (Kaddumukasa et al., 2016), while another reports a crude prevalence of 7–20 per 100,000 (Blanckenberg, Bardien, Glanzmann, Okubadejo, & Carr, 2013). African Americans are diagnosed with PD less frequently than Caucasians, independent of age, sex, income, or insurance differences (Dahodwala et al., 2009), suggesting a genetic component.

While multiple SNPs have been implicated in *SNCA*‐related phenotypes, the genetics of *SNCA* remains uncertain at present, further confounded by the likely presence of more than one functional variant. A sound interpretation of clinical associations requires a better understanding of the underlying molecular mechanism to resolve the overall influence of genetic *SNCA* variation and its impact across populations.

Rhinn et al. (2012) have performed studies on expression of short (290, 480, and 560 bp) and extended length (1,070 and 2,520 bp) 3′UTR SNCA isoforms and characterized variants (including rs356165) associated with 3′UTR length and function. Using a short isoform of the SNCA 3′UTR (574 bp), it was reported that rs17016074, NM_000345.3(SNCA):c.*501C>T, moderately enhances expression in a luciferase reporter gene assay (Sotiriou, Gibney, Baxevanis, & Nussbaum, 2009). In contrast, others measured SNCA and searched for SNPs in the 3′UTR associated with mRNA expression (Linnertz et al., 2009). Their results suggested reduced mRNA expression associated with the minor *A* allele of rs17016074. Others (Junn et al., 2009; Kabaria et al., 2015) have demonstrated the impact of miRs on alpha‐synuclein, studying the relevance of 3′UTR isoforms, including a reporter gene assay with a full length rat 3′UTR (Kabaria et al., 2015). Yet, the molecular genetics of *SNCA* remains incompletely understood. In this study, we search for regulatory variants across the *SNCA* locus, affecting transcription, RNA processing, and translation including a comparison between full length (2,520 bp) and shorter (574 or 1,070 bp) 3′UTR isoforms. The approach involves measurements of allelic mRNA expression differences revealing allelic expression imbalance (AEI) in human brain autopsy tissues as an indicator of *cis*‐acting regulatory effects (Moyer et al., 2011; Smith et al., 2013; Wang, Para, Koletar, & Sadee, 2011).

## 2 MATERIALS AND METHODS

### 2.1 Ethical compliance

This study has been approved by an ethics committee. It was determined that the work did not require full review as it did not meet the federal definition of human subjects research because the study was limited to deidentified autopsy tissues.

### 2.2 Sample preparation

DNA (Miller, Dykes, & Polesky, 1988) and RNA (Wang, Johnson, Papp, Kroetz, & Sadee, 2005) were isolated from flash fresh‐frozen human postmortem brain tissue samples. Human prefrontal cortex (Brodmann area 46) samples (*N* = 215) were provided by the Miami Brain Endowment Bank (University of Miami, Miami, FL, USA): 184 from males and 31 from females. There were 76 African Americans and 139 Caucasians; 55 were also Hispanic. The mean age was 36.1 ± 10.3. A total of 12 anterior cingulate cortex samples from eight subjects with pure Lewy body dementia and four subjects with mixed Alzheimer's disease and Lewy body dementia were provided by the Ohio State University (OSU) Neurodegenerative Disease Brain Tissue Repository (Buckeye Brain Bank, USA). All were Caucasian, with the mean age 75.3 ± 7.4. There were eight males and four females. cDNA was prepared from total RNA via reverse transcription with SuperScript III (Invitrogen, Waltham, MA, USA), using oligo‐dT and gene‐specific primers.

### 2.3 Quantitative real‐time reverse transcription PCR (qRT‐PCR)

SNCA mRNA expression was measured on the 7,500 Fast Real‐Time PCR system (Life Technologies, Oyster Point, CA, USA), using Fast SYBR Green Master Mix (Life Technologies). Samples were run in duplicate and normalized to the housekeeping gene *PGK1*. *SNCA* primer sequences are available in Table S1.

### 2.4 Genotyping

The GenBank reference for *SNCA* is NC_000004.11. Individual genotyping was performed with a fluorescent restriction fragment length polymorphism assay, allele‐specific melting curve analysis (Papp, Pinsonneault, Cooke, & Sadee, 2003), or the SNaPshot primer extension assay described below.

### 2.5 Allelic mRNA expression analysis

A SNaPshot primer extension assay (Life Technologies) was used to measure allelic mRNA ratios in heterozygous carriers of a marker SNP in the transcribed region (Moyer et al., 2011; Smith et al., 2013; Wang et al., 2011). Two marker SNPs were used: rs356165 and rs17016074. A region containing the marker SNP is PCR amplified, followed by a single base extension reaction to add a fluorescently labeled dideoxynucleotide complementary to the SNP, and allelic ratios are quantitated on a 3,730 DNA Analyzer capillary electrophoresis instrument (Life Technologies). Measured after conversion to cDNA, the allelic mRNA ratios are normalized to the mean of the measured genomic DNA ratios.

### 2.6 Reporter gene assay to test rs17016074 activity

A region (3,794 bp) surrounding the full length 3′UTR was PCR amplified from DNA samples homozygous for either the major or minor allele of rs17016074 (primer sequences in Table S1). The product was reamplified using primers designed for the In‐Fusion HD Eco Dry cloning kit (Clontech, Mountain View, CA, USA) specific to the full length 3′UTR (2,529 bp). The amplicon was then inserted into a modified version of the luciferase reporter gene vector pGL3 Basic (Promega, Madison, WI, USA), containing a CMV promoter upstream of the luciferase gene. The amplicon was inserted immediately 3′ of the luciferase coding region, utilizing the restriction sites Xba1 and BamH1, which removes the SV‐40 polyadenylation site from the vector. All constructs were sequenced to confirm fidelity. Plasmids were transfected into SH‐SY5Y cells with a Renilla‐expressing control vector in 12‐well plates utilizing lipofectamine 2000. Cells were cultured at 37°C in a humidified incubator with 5% CO_2_ in DMEM/F12 and 10% fetal bovine serum and 1× Pen/Strep. Activity was measured with Dual‐Glo reagents (Promega) on a fluorescence plate reader (PerkinElmer Life and Analytical Science, Waltham, MA, USA). To measure allelic ratios of the 3ʹUTR expression constructs, we cotransfected equal amounts of plasmids with either the *G* or *A* allele of rs17016074. Cells were harvested 24, 48, or 72 hrs posttransfection. DNA and RNA were isolated and allelic ratios measured as described above.

### 2.7 Genome‐wide association studies (GWAS)

Genome‐wide association studies datasets to compare rates and risk factors for dementia generated by other institutions were downloaded from dbGaP. The “Genome Wide Association study of Yoruba in Nigeria” is part of the “Indianapolis – Ibadan Dementia Project (R01 AG009956)” funded by the Division of Neuroscience, National Institute on Aging, National Institutes of Health; it was downloaded from dbGaP phs000378.v1.p1. This project is compliant with the regulations of the OSU Institutional Review Board (IRB) and operates under a protocol approved by a duly constituted ethics committee (OSU IRB).

## 3 RESULTS

Use of AEI ratios sensitively detects the magnitude of allelic effects on expression of mRNA and its isoforms, while also enabling scanning the gene locus for the responsible *cis‐*acting regulatory variants. We determine here that rs17016074* G>A* alters SNCA 3′UTR isoform expression and affects translation. We also confirm that the rs356165 *A* allele enhances mRNA expression.

### 3.1 Allelic SNCA mRNA ratios as an indicator of regulatory polymorphisms

Using RNA extracted from human prefrontal cortex samples, we measured allelic mRNA expression ratios at marker SNP rs356165 located in the 3′UTR downstream of polyadenylation sites generating short 3′UTR isoforms (Figure S1). We did not detect evidence of copy number variation (gene duplications) in the gDNA allelic ratios. Testing additional variables revealed no effect of sex (*p* = .44) or age (*p* = .87) on allelic ratios.

**Figure 1:**
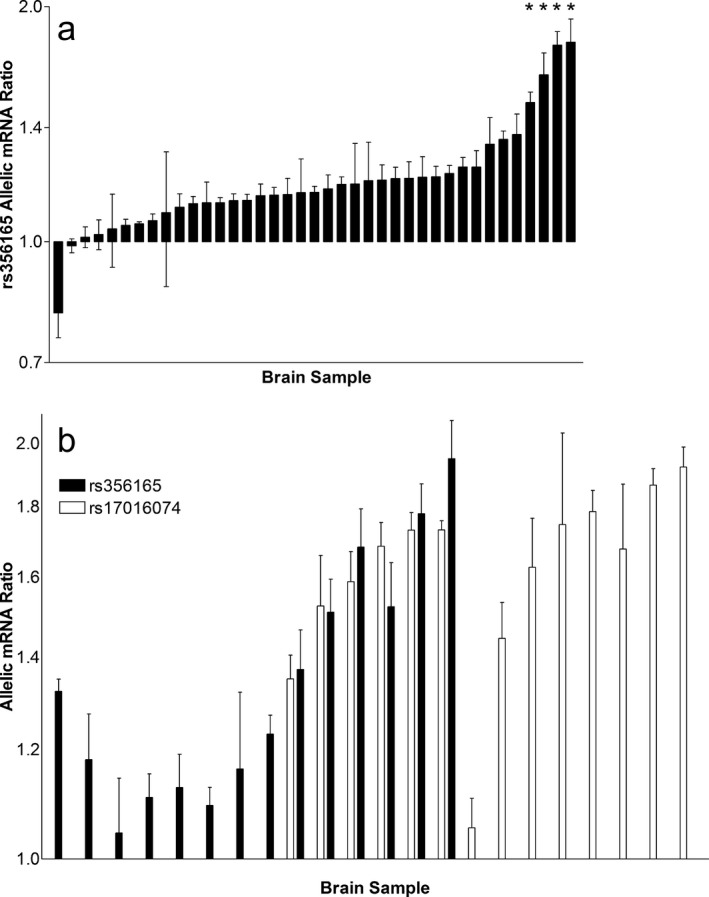
Allelic SNCA mRNA expression ratios in human brain tissues. (a) Allelic ratios were measured in heterozygous carriers of marker rs356165, located in the long 3′UTR. Samples marked with * are heterozygous for rs17016074. (b) Allelic ratios were obtained in heterozygous carriers at both markers rs356165 (black bars) and rs17016074 (open bars). Peak heights ratios are standardized to the measured genomic DNA allelic ratio. Each bar is the mean from one subject (*n* = 4, ±*SEM*). All AEI ratios measured at rs356165 (major/minor) were <1 (with the exception of one sample) so the inverse value (minor/major) is plotted for ease of comparison to AEI measured at rs17016074, with its minor allele on the opposite strand. Allelic ratios at rs17016074 were obtained with use of PCR‐extended amplicons specific for the long 3′UTR; short amplicons measuring both short and long 3′UTR did not reveal large AEI (see Figure S2)

Four tissues demonstrated robust AEI measured at rs356165 (Figure 1a, far right), all with ratios in the same direction, indicating that the functional SNP is likely in LD with this marker. We then genotyped *SNCA* SNPs previously identified in GWAS, haplotype‐tagging SNPs, and those located in close proximity (Table 1). All four samples with robust AEI were heterozygous for rs17016074, whereas all remaining samples were homozygous, sufficient to implicate rs17016074 as a causative candidate variant with the lowest *p* value (*p* = .00003) (Table 1)., Heterozygous carriers in our tissue samples are primarily from African Americans.

**Table 1:** SNPs genotyped and genotype association with AEI using the *F* statistic

Marker	*F*‐Test *P*	Bonferroni *P*
rs17016074	0.000031	0.0008
rs7678651	0.018	0.48
rs11097234	0.018	0.48
rs3857059	0.056	1
rs3775446	0.23	1
rs356221	0.25	1
rs2583958	0.27	1
rs2583985	0.27	1
rs2583988	0.27	1
rs2619364	0.27	1
rs2736995	0.27	1
rs2737006	0.27	1
rs2736990	0.42	1
rs356200	0.48	1
rs974711	0.48	1
rs1812923	0.48	1
rs2301134	0.48	1
rs7684318	0.48	1
rs7687945	0.48	1
rs2583988	0.57	1
rs356219	0.71	1
rs748849	0.71	1
rs2197120	0.71	1
rs2737030	0.71	1
rs2619363	0.73	1

As rs17016074 is located in the 3′UTR more proximal than rs356165 within short 3′UTR isoforms, it was also used as a marker SNP to measure AEI. However, pronounced imbalance was not observed in these PCR products, and the AEI ratios detected at rs17016047 were discrepant from those at rs356165 in tissues heterozygous for both (Figures 1a, S2).

To test whether these differences in AEI results between the two markers are due to changing an intervening polyadenylation site, we amplified a 667 bp region containing both rs356165 and rs17016074, in 22 brain samples, including all samples heterozygous for rs17016074 (*n* = 14). The PCR product encompassing both SNPs specifically amplifies only long 3′UTR isoforms. AEI was measured at both SNPs (Figure 1b) when the sample was a compound heterozygote (middle section), rs356165 alone (far left) or rs17016074 alone (far right) (Figure 1b). With use of this longer amplicon, the discrepancy between AEI ratios measured at the two marker SNPs is resolved, yielding similar ratios (*R* ^2 ^= .65). Significant AEI was measured in all samples at rs17016074 (with one exception). As the allelic ratio is >1 (*G* allele/*A* allele), the major *G* allele of rs17016074 associates with higher mRNA expression (residing on the same haplotype as the *A* allele of rs356165).

To search for other variants in high LD with rs17016047, we extracted all SNPs with *R* ^2^ > .8 from the SNAP database (http://archive.broadinstitute.org/mpg/snap/) (using hg18 coordinates). There were no results for the European cohort, while in the African (YRI) population, 16 SNPs spread over 46 kb had an *R* ^2^ of 1.0, and 68 SNPs with an *R* ^2 ^> .885 (Table S2). The exceptionally long haplotype with a frequency of 13% suggests evolutionary selection on the basis of its genetic influence. The closest SNP in high LD with rs17016047 is rs6842093. Given the location of rs6842093, 587 bp 5′ of rs17016047 in an intron within the coding region of *SNCA*, it is unlikely to alter polyadenylation site usage.

### 3.2 rs17016074 genotype association with 3′UTR length

To quantify the relative expression of the long 3′UTR, we used qRT‐PCR to measure expression of the long 3′UTR isoforms in prefrontal cortex brain tissues, normalized to overall expression of SNCA mRNA (Figure S3). The long 3′UTR expression was significantly higher in the homozygous major (*GG*) compared to the heterozygous (*GA*) samples (*p* = .03). This result indicates that the minor *A* allele enhances usage of a more proximal polyadenylation site leading to lower expression of the long 3′UTR isoforms, and by inference from the allelic RNA ratios at rs356165, higher expression of a short 3ʹUTR isoform.

### 3.3 Effect of rs17016047 on luciferase 3′UTR reporter gene activity and mRNA expression in transfected cells

**Figure 2:**
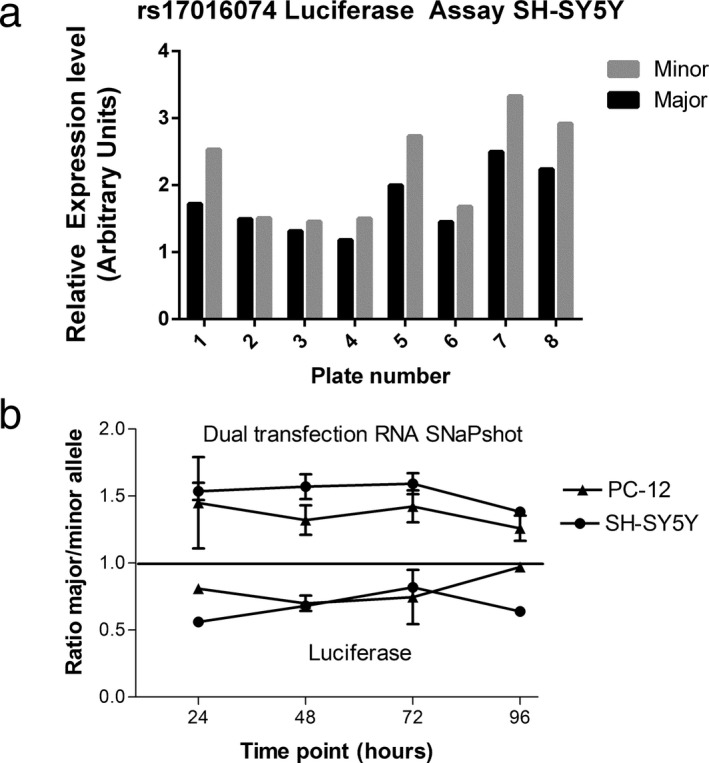
Effect of rs17016074 alleles on in vitro expression. (a) Translational activity of the long *SNCA* 3′UTR containing either the major (*G*) or minor (*A*) allele measured in a luciferase reporter gene assay in SH‐SY5Y cells. A ratio of major/minor allele <1 indicates higher protein activity of the minor allele (paired *t*‐test *p* = .02; eight independent experiments). (b) Allelic mRNA expression ratios (major/minor alleles) of SNCA over time in PC‐12 and SH‐SY5Y cells after cotransfection of equal amounts of rs17016074 major *G* and minor *A* allele constructs of full length 3′UTR. A ratio above one indicates higher RNA expression of the major *G* allele construct. Translational activity is shown in the lower portion of the graph

While previous 3′UTR reporter gene assays had used only various short to medium length human SNCA 3′UTRs (560–1,100 bp) (Rhinn et al., 2012), we inserted the annotated full length 3′UTR (2,520 bp). First, we compared the minor and major allele of rs170160174 to determine whether this SNP affects luciferase expression in SH‐SY5Y cells (human neuroblastoma). We consistently observed more luciferase activity of the minor versus the major allele across eight replicates (Figure 2a), resulting in a major/minor allele activity ratio <1. This result is consistent with results obtained from 3ʹUTR constructs of a short isoform (574 bp) carrying the *G* or *A* allele of rs17016074 (Sotiriou et al., 2009). Testing the kinetics of allelic expression, the allelic differences remained similar at various time‐points after transfection, from 24 to 96 hrs. Similar results were obtained in PC‐12 (rat pheochromocytoma) (not shown). Next, we cotransfected the vectors carrying the major and the minor allele in equal amounts, in the same two cell lines, harvested RNA and measured allelic ratios at marker rs356165 with SNaPshot as previously described. In contrast to the luciferase activity results, the major *G* allele of rs17016074 generated higher mRNA levels than the minor allele.

We also tested the effects of reporter gene vectors carrying different lengths of the 3′UTR. The ratio between major and minor alleles was more pronounced in the full construct (2,520 bp) compared to shorter 3′UTRs of 574 or 1,070 bp. In all experiments, the minor *A* allele was more productive than the major *G* allele (~1.25 ratio for full length, ~1.18 ratio for shorter constructs), consistent with previous data (Sotiriou et al., 2009), but the difference was not large enough to be statistically significant. Taken together, the results show that the minor allele is associated with lower long 3′UTR mRNA expression but higher translation of the luciferase reporter activity.

### 3.4 Association of rs356165 with AEI independent of large allelic ratios

Tissue samples homozygous for rs17016047 exhibit moderate AEI (Figure 1a), indicating the presence of a more frequent SNP with a smaller effect on allelic mRNA ratios. Comparing the allelic gDNA and cDNA ratios at rs356165, excluding those also heterozygous for rs17016047, revealed a significant difference (*p* = 8.8 × 10^−6^), establishing the presence of AEI indicative another regulatory genetic factor (Figure S4). The marker SNP rs356165 itself is consistently associated with allelic imbalance, with minor/major allele ratios above 1, indicating that the minor allele is associated with higher mRNA expression (Figure 1a). Use of allelic ratio analysis in this study reveals a *cis‐*eQTL effect of rs356165 on mRNA expression in prefrontal cortex and anterior cingulated cortex, but at a level likely not detectable as an eQTL using overall mRNA expression. Therefore, rs356165 (or variants in high LD) is a candidate regulatory variant affecting SNCA mRNA expression in the brain.

With a minor allele frequency (MAF) of 36% in Europeans, 66% in Africans, rs356165 also resides in a long haplotype spread across >30 kb (Table S3). While rs356165 has been implicated previously in PD, we cannot rule out other variants in this high LD block as causative regulatory factors.

### 3.5 mRNA folding analysis

**Figure 3:**
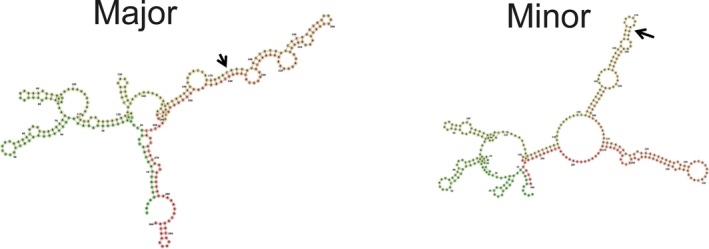
Predicted RNA folding with either the major (left) or minor allele (right) of rs17016074 for a 300 bp region surrounding the SNP. Structures are obtained from the RNAfold server. http://rna.tbi.univie.ac.at/cgi-bin/RNAfold.cgi?PAGE=3&ID=hwOIKm8pZk

We tested the effect of rs17016074 on the secondary structure of SNCA mRNA, using the RNAfold program. For a localized analysis and illustration purposes, we included a 300 bp region surrounding the SNP (Figure 3). Substitution of a *T* in place of *C*, resulted in a clear difference between the folding configurations. Substantial folding changes are also observed when including the entire 3′UTR. For the thermodynamic ensemble prediction, the minimum free energy for the secondary structure changed from −483.99 kcal/mol with the major allele, to −391.57 kcal/mol with the minor allele. These results indicate that rs1706074 can affect the secondary structure of the 3′UTR. Another mechanism could involve alteration of binding motifs: rs17016074 alters a transcriptional repressor motif (CTCF also known as 11‐zinc finger protein) (HaploReg v4.1).

### 3.6 GWAS analysis of rs1706074, rs356165, and other candidate SNPs

Our results suggest the presence of at least two regulatory variants within *SNCA*, rs17016074 and another marked by rs356165. We tested the effects of these two variants on memory in an African cohort from “Genome Wide Association study of Yoruba in Nigeria.” In this Yoruban population, the MAFs for rs17016074 and rs356165 are 24% and 16%, respectively. Memory was assessed as either immediate recall or delayed recall. rs17016047 failed to show a significant detectable effect on memory functions (*p* = .58 for the immediate recall, *p* = .81 for delayed recall). Although not individually significant, rs17016074 could interact with other causative variants. On the other hand, rs356165 was associated with reduced immediate recall and delayed recall scores with a *p* value of 6.3e‐4 (effect size = −0.18) and 1.5e‐2 (effect size = −0.18), respectively.

To evaluate the combined effect of rs17016074 and rs356165, we built haplotypes of the two variants and examined association of each haplotype with the immediate and delayed recall scores. Because of the LD structure of the two variants, only three haplotypes (rs17016074 – rs356165: *GG, GA, AG*) were found. Among the three, the *GA* haplotype is significantly associated with immediate and delayed recall conveying deteriorating effect on memory performance (Table 2); however, the results are insufficient to support an independent effect of rs17016074 on cognitive functions.

**Table 2:** Frequency of rs356165 (G>A) and rs1716074 (G>A) haplotypes and their association with immediate and delayed recall scores. The AA haplotype is rare, as these two SNPs reside on opposite alleles

Haplotype rs356165/rs17016074	Frequency	Immediate recall (effect size)	Delayed recall (effect size)
AG	0.24	−0.21 (*p* = .004)	−0.17 (*p* = .011)
GA	0.16	−0.09 (*p* = .27)	−0.07 (*p* = .41)
GG	0.60	Baseline	Baseline

## 4 DISCUSSION

The results of this study identify the presence of at least two *cis*‐acting regulatory variants affecting SNCA mRNA expression, as identified by measurement of allelic ratios in the 3′UTR. We demonstrate that a large allelic imbalance is associated with rs17016074, while moderate allelic ratios occur in the presence of rs356165, located in an extended block of SNPs in high LD. Detailed analysis in luciferase reporter gene assays of the SNCA 3′UTR, incorporating the long full length annotated 3′UTR, revealed that the rs17016074 minor *A* allele is associated with lower mRNA transcript expression but higher expression of luciferase protein activity, demonstrating opposite effects in these in vitro assays, and reconciling seemingly contradictory results on rs17016074 (Linnertz et al., 2009; Sotiriou et al., 2009).

In previous studies, the minor *A* allele of rs17016074 had also been tentatively associated with lower SNCA mRNA levels in human tissues (Linnertz et al., 2009), whereas the minor allele caused increased luciferase expression in transfection experiments with a short isoform of the 3ʹUTR in SH‐SY5Y cells (Sotiriou et al., 2009). As reporter gene assays may not represent the effect in intact tissue, we first used allelic ratio analysis in brain autopsy tissues, showing that the minor allele of rs17016074, located between two polyA signals, decreases formation of the long 3′UTR isoforms and in turn increases the short 3′UTR. Because the amplicon used for the AEI analysis at rs17016047 encompasses both the long and short 3′UTR isoforms, the variant presumably alters polyadenylation site usage, as previously demonstrated with a 3′UTR SNP in *NAT1,* if long and short 3′UTR isoforms have similar turnover rates (Wang et al., 2011).

Sotiriou et al. (2009) had observed enhanced protein expression in a luciferase 3ʹUTR reporter gene assay using only the short 3ʹUTR (574 bp) suggesting that rs17016074 might have a dual action: enhancing formation of the short 3ʹUTR isoforms and further enhancing translation from the short 3ʹUTR. Several miR target sites along the SNCA 3′UTR add an additional mechanisms by which rs17016074 can affect alpha‐synuclein expression *via* altered interaction with miR7 and miR24b,c; the miR24c target site resides in a more distal long 3′UTR location (Junn et al., 2009; Kabaria et al., 2015). These miRs are downregulated, in concert with alpha‐synuclein being upregulated, while additional miRs have also been shown to be dysregulated in PD (Tatura et al., 2016).

We propose that the short 3′UTR supports more efficient translation than the long 3′UTR, with rs17016074 altering regulatory functions of the 3′UTR. This result is in agreement with a recent report that *SNCA* with a short 3′UTR (575 bp) supports the highest protein expression in vitro compared to medium (1,074 bp) and full length (2,530 bp) UTRs (Marchese et al., 2017). The study also identified an RNA‐binding protein, TIAR, that interacts with the *SNCA* 3′UTR; rs17010674 is located in one of its binding sites and has a PHRED score >10, supporting a proposed effect on SNCA expression (Marchese et al., 2017).

Previous reports have demonstrated an effect on RNA folding by rs356165 (Rhinn et al., 2012), which may alter mRNA expression indicated by the moderate AEI ratios observed at this marker. These results support possible regulatory functions of both rs17016047 and rs356165 at the level of RNA structure.

Taken together, our results firmly implicate rs17016074 as a regulatory variant affecting SNCA mRNA isoform expression. It remains to be determined whether the net effect in vivo is an increase or decrease in SNCA protein expression. As the processes involved in protein expression are under the control of multiple factors, and likely differ between brain regions, it is also possible that rs17016074 could have variable effects under different conditions, and may therefore be detectable in GWAS only for specific phenotypes or under specific conditions. This study includes AEI ratio measurements of a small number of brain tissues from DLB patients, to address any impact of pathophysiology of SNCA expression. While taken from a different brain region (anterior cingulate cortex vs. Brodmann area 46), rs356165 displays similar allelic mRNA ratios in the DLB patients, but the number of samples is insufficient to reveal small changes in DLB. One of these samples is heterozygous for rs17016074, and it also displays robust AEI, as expected (Figure 1a). However, a large study is needed to address any genetic effects of rs1701607 and rs365165 that are region‐selective or altered by pathophysiology.

Of note, the minor *A* allele of rs17016074 resides on the same haplotype as the major (protective) *G* allele of rs356165, which might mask any deleterious effect of rs17016074. Given the robust effect of rs17016074 on allelic expression of the long 3′UTR isoform and its high MAF in Africans (13% vs. 0.1% in Europeans, gnomad.broadinstitute.org), it remains to be determined whether rs17016074 could have a protective or deleterious effect.

An *SNCA* region in intron 4 carries variants in high LD with rs17016074 in subjects of African descent. In populations of European ancestry, one intronic SNP, rs17016126, occurs at a large distance (45,326 bases from rs17016074) but is in high LD with rs6842093 (a proxy for rs17016074 in Europeans), D′ = 1 and *R* ^2* *^= 1 (Africans, D′ = 0.96, *R* ^2 ^= .80). As the genetic effect appears to be differential formation of 3′UTR isoforms, it is unlikely that this event could have been caused by a remote variant, but the presence of additional functional variants on this haplotype cannot be excluded. The length of these LD blocks of varying frequencies among ethnic groups is an indicator of evolutionary selection pressure.

With rs356165 embedded in a long LD block, it is not possible to firmly assign the causative variant. Linnertz et al. (2009) have demonstrated enhanced mRNA expression in autopsy brain tissues of neurologically normal subjects to be associated with homozygous carrier status for the minor *A* allele of rs356165, consistent with results in this study showing increased mRNA expression by measuring allelic mRNA ratios. While reduced cognitive function is consistent with enhanced SNCA expression, rs356156 has been implicated as a protective factor against PD (Linnertz et al., 2009), an unresolved discrepancy.

In case–control comparisons in European populations, the *G* allele of rs356165, located in the 3ʹUTR, represents a risk allele for PD, with the minor *A* allele being protective (Cardo et al., 2012; Mizuta et al., 2006; Mueller et al., 2005; Myhre et al., 2008; Ross et al., 2007; Schmitt et al., 2012; Winkler et al., 2007). However, no association was found between rs356165 genotype and PD in Chinese subjects (Hu et al., 2010). rs17016074 and rs356165 are in high LD with each other (D′ = 0.99) but with low *R* ^2^ (.05 to .09) residing on opposite DNA strands (phased 1,000 Genomes data, depending on population). For example, a carrier heterozygous for both SNPs would have the genotype *GA* for rs356165, and *AG* for rs17016074, but *AA* haplotypes are rare. This relationship needs to be considered in clinical association studies when the occurrence of minor alleles is not independent of each other.

As rs17016074 is relatively frequent in African Americans, we initiated a study of its possible association with cognitive functions and PD. However, most GWAS studies focus on Caucasians, limiting our ability to test the effect of rs17016074. In the “Genome Wide Association study of Yoruba in Nigeria” we confirm association of rs356165 (with a MAF of 24% in this population) with cognitive functions, consistent with allelic differences in mRNA expression. In Yorubans, only a few SNP located around the 3′UTR are in high LD (*R* ^2^) with rs356165, suggesting the causative variant resides in this region. On the other hand, we do not find sufficient evidence for an association of rs17016074 with cognitive functions, either alone or in a haplotype with rs356165. Assessing the overall impact of *SNCA* polymorphisms will require large‐scale studies, addressing dynamic interactions between *SNCA* variants. Further analysis is required to determine whether rs17016074 affects cognitive functions or risk of neurodegenerative disorders, and under what conditions.

The large difference in rs1706074 allele frequencies between Caucasians and Africans and differences in specific neurodegenerative disease rates could provide hints as to underlying genetic factors, such as the lower incidence of PD in African Americans compared to Europeans (Dahodwala et al., 2009). On the other hand, a larger proportion of African Americans have Lewy Bodies and infarction co‐occurring with Alzheimer's disease compared to Caucasians (Barnes et al., 2015). Among older adults, African Americans are more likely than older Caucasians to have Alzheimer's and other dementias (Manly & Espino, 2004; Potter et al., 2009). Future studies should address these ethnic differences to enhance our understanding of the neurodegenerative processes involving *SNCA* and determine our ability to intervene or prevent disease more effectively.

## CONFLICT OF INTEREST DISCLOSURE

The authors have no conflicts of interest to disclose.
