# Supplementary material for: Alpha‐synuclein mRNA isoform formation and translation affected by polymorphism in the human SNCA 3ʹUTR
Source: Mol Genet Genomic Med. 2018 May 6;6(4):565–74. doi: 10.1002/mgg3.407 (PMC6081226; doi:10.1002/mgg3.407)
Supplement: Supplementary file 1 [file MGG3-6-565-s001.docx]

SUPPLEMENT

**Supplemental Table 1**. Sequences of primers used

| **Purpose** | **F/R** | **Primer Sequence** |
| --- | --- | --- |
| qPCR | F | AATACTTAAAAATATGTGAGCATGAAACTATG |
|  | R | TTTATTTTAATTCTCACCATTTATATACAAACAC |
| rs356165 RFLP | F | CAGCATTCACACCAATATCAGACA |
|  | R | GAATTCCCTGAAGCAACACTGC |
|  | R_FAM | [6FAM]GAATTCCCTGAAGCAACACTGC |
| rs356165 SNaPshot | F | CAGCATTCACACCAATATCAGACA |
|  | R | GAATTCCCTGAAGCAACACTGC |
|  | PEF | AACAACAGTTCCCCAAAATAC |
| rs17016074 SNaPshot | F | CTCACCATTTATATACAAACACAAGTGAAT |
|  | R | TCTTTTAATGATACTGTCTAAGAATAATGACG |
|  | R_alt | GTATCTGTACCTGCCCCCACTC |
|  | PEF | AACACAAGTGAATAAAACACATC |
| SNaPshot both SNPs | F | CAGCATTCACACCAATATCAGACA |
|  | R | TCTTTTAATGATACTGTCTAAGAATAATGACG |
| rs17016074 allele-specific genotyping | F_G | CAAACACAAGTGAATAAAACACTTCG |
|  | F_Agc | GCCCGCCGCCCCCGCAAACACAAGTGAATAAAACACACCA |
|  | R | AATACTTAAAAATATGTGAGCATGAAACTATG |
| Reporter gene PCR | F | AATGTCAGCCCTGTTGCACT |
|  | R | GCTGTCAGTGCTGATGCGTA |
| Infusion PCR | F | GCCGTGTAATTCTAGAGAAATATCTTTGCTCCCAG |
|  | R | ATCGGTCGACGGATCGAGGCAGAAACCAAATACAT |
| 1070 bp UTR | R | GTCGAATATTATTTATTGTCAGAAAGGTACAGCATTCACA |
|  | infR | ATCGGTCGACGGATCCGTCGAATATTATTTATTGTCAG |
| 575 bp UTR | R | TATTTTTGCAATGAGATAACGTTTTATTTTAATTCTCACCA |
|  | infR | ATCGGTCGACGGATCCTATTTTTGCAATGAGATAACGT |

**Supplemental Table 2**. SNPs in high LD (R^2^>0.9) with rs17016074 in YRI (African) population. Only limited data are available for CEU (Caucasian) and JPT/CHB (Japanese/Chinese)

| Proxy | Distance | R^2^ | Dʹ | Coordinate_HG18 | MAF |
| --- | --- | --- | --- | --- | --- |
| rs17016074 | 0 | 1 | 1 | 90866301 | 0.14 |
| rs6842093 | 587 | 1 | 1 | 90866888 | 0.14 |
| rs116451921 | 2089 | 1 | 1 | 90868390 | 0.14 |
| rs34351491 | 4284 | 1 | 1 | 90870585 | 0.14 |
| rs17016083 | 5129 | 1 | 1 | 90871430 | 0.14 |
| rs76648536 | 8871 | 1 | 1 | 90875172 | 0.14 |
| rs76223274 | 9839 | 1 | 1 | 90856462 | 0.14 |
| rs3857054 | 13192 | 1 | 1 | 90879493 | 0.14 |
| rs6532188 | 17344 | 1 | 1 | 90848957 | 0.14 |
| rs34747217 | 18974 | 1 | 1 | 90885275 | 0.14 |
| rs74714220 | 19578 | 1 | 1 | 90885879 | 0.14 |
| rs57928386 | 21322 | 1 | 1 | 90887623 | 0.14 |
| rs57267227 | 21532 | 1 | 1 | 90887833 | 0.14 |
| rs74539083 | 21736 | 1 | 1 | 90888037 | 0.14 |
| rs80062285 | 22372 | 1 | 1 | 90888673 | 0.14 |
| rs35771728 | 28615 | 1 | 1 | 90894916 | 0.14 |
| rs6814056 | 17397 | 0.932 | 1 | 90848904 | 0.14 |
| rs6532187 | 18171 | 0.932 | 1 | 90848130 | 0.14 |
| rs6532186 | 18289 | 0.932 | 1 | 90848012 | 0.14 |
| rs6532185 | 18441 | 0.932 | 1 | 90847860 | 0.14 |
| rs6818294 | 4442 | 0.928 | 1 | 90861859 | 0.13 |
| rs79613539 | 8044 | 0.928 | 1 | 90874345 | 0.13 |
| rs116167791 | 17795 | 0.928 | 1 | 90884096 | 0.13 |
| rs4635786 | 24244 | 0.928 | 1 | 90890545 | 0.13 |
| rs114989493 | 32535 | 0.928 | 1 | 90898836 | 0.13 |
| rs77141123 | 32742 | 0.928 | 1 | 90899043 | 0.13 |
| rs35921673 | 36391 | 0.928 | 1 | 90902692 | 0.13 |
| rs58877857 | 39856 | 0.928 | 1 | 90906157 | 0.13 |
| rs17016124 | 43621 | 0.928 | 1 | 90909922 | 0.13 |
| rs17016126 | 45913 | 0.928 | 1 | 90912214 | 0.13 |
| rs75302617 | 47013 | 0.928 | 1 | 90913314 | 0.13 |
| rs78601023 | 47333 | 0.928 | 1 | 90913634 | 0.13 |
| rs76232707 | 47413 | 0.928 | 1 | 90913714 | 0.13 |
| rs77504605 | 47781 | 0.928 | 1 | 90914082 | 0.13 |
| rs114922311 | 47992 | 0.928 | 1 | 90914293 | 0.13 |
| rs10516846 | 49057 | 0.928 | 1 | 90915358 | 0.13 |
| rs6825073 | 49474 | 0.928 | 1 | 90915775 | 0.13 |
| rs114350019 | 50448 | 0.928 | 1 | 90916749 | 0.13 |
| rs114203365 | 54296 | 0.928 | 1 | 90920597 | 0.13 |
| rs3775435 | 55751 | 0.928 | 1 | 90922052 | 0.13 |
| rs3822088 | 56715 | 0.928 | 1 | 90923016 | 0.13 |
| rs3796662 | 57277 | 0.928 | 1 | 90923578 | 0.13 |
| rs3775437 | 58665 | 0.928 | 1 | 90924966 | 0.13 |
| rs3775438 | 60625 | 0.928 | 1 | 90926926 | 0.13 |
| rs3822091 | 62760 | 0.928 | 1 | 90929061 | 0.13 |
| rs3775442 | 67953 | 0.928 | 1 | 90934254 | 0.13 |
| rs34683772 | 78258 | 0.928 | 1 | 90944559 | 0.13 |
| rs35540244 | 78260 | 0.928 | 1 | 90944561 | 0.13 |
| rs961386 | 85525 | 0.928 | 1 | 90951826 | 0.13 |
| rs3775450 | 87636 | 0.928 | 1 | 90953937 | 0.13 |

**Supplemental Table 3**. SNPs in high LD (R^2^>0.9) with rs356165 in CEU, YRI and JPT/CHB populations

**CEU**

Proxy Distance R^2^ Dʹ Coordinate_HG17 MAF

rs356220 5546 1.000 1.000 90998518 0.43

rs356219 9285 0.967 1.000 90994779 0.42

**YRI**

Proxy Distance R^2^ Dʹ Coordinate_HG18 MAF

rs356220 5546 0.957 1.000 90860363 0.25

**JPT+CHB**

Proxy Distance R^2^ Dʹ Coordinate_HG18 MAF

rs8180214 2378 0.978 1.000 90863531 0.44

rs8180209 2432 0.978 1.000 90863477 0.44

rs3775423 10605 0.978 1.000 90876514 0.44

rs7661330 16784 0.978 1.000 90882693 0.44

rs3775424 18370 0.978 1.000 90884279 0.44

rs3756054 27565 0.978 1.000 90893474 0.44

rs3857059 28352 0.978 1.000 90894261 0.44

rs3857052 3029 0.956 1.000 90862880 0.45

rs10003708 3835 0.956 1.000 90862074 0.45

rs7655792 4861 0.956 1.000 90861048 0.45

rs356220 5546 0.956 1.000 90860363 0.45

rs11931074 7371 0.956 1.000 90858538 0.45

rs7681815 8272 0.956 1.000 90857637 0.45

rs7681312 8359 0.956 1.000 90857550 0.45

rs356219 9285 0.956 1.000 90856624 0.45


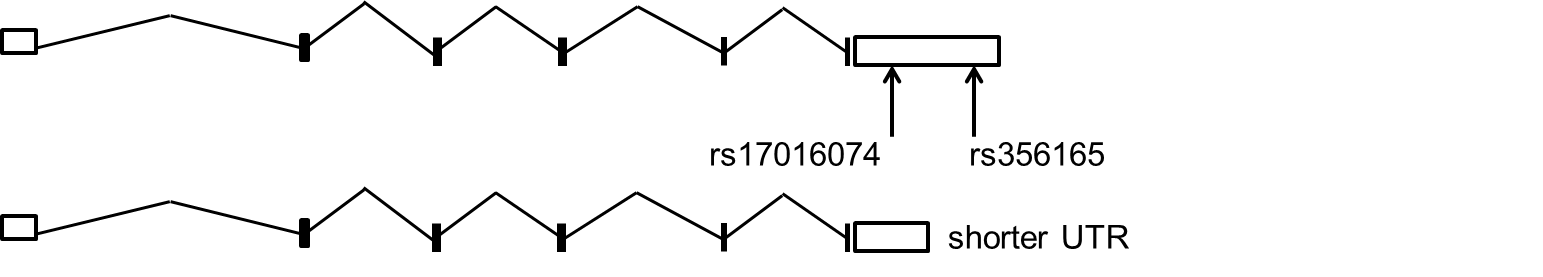


Figure S1. Gene map with marker SNPs. Distances and SNP locations are approximate. Filled boxes indicate exons and open boxes indicate UTRs (untranslated regions).


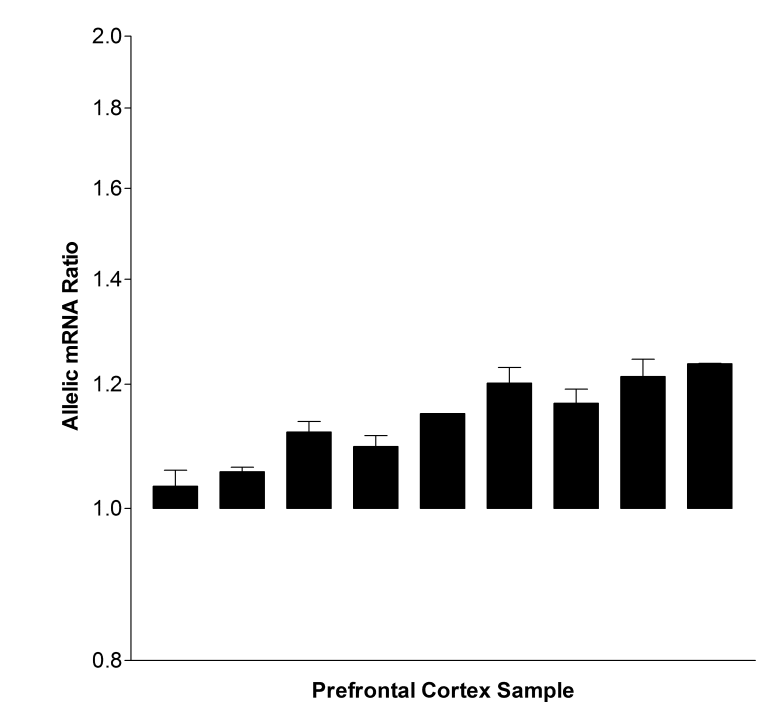


Figure S2. Allelic SNCA mRNA expression ratios measured at marker rs17016074 in brain cDNA samples. Primers amplify a short PCR product encompassing both 3′UTR lengths. The values are plotted as peak height ratios of the major (*G*) over the minor (*A*) allele, standardized to the measured genomic DNA allelic ratio. All samples are heterozygous for rs17016074, serving as the marker SNP. Each bar is the mean AEI measurement from one subject (n=2). The observed deviation from unity is largely attributable to AEI associated with rs356165 (see Figure S4).


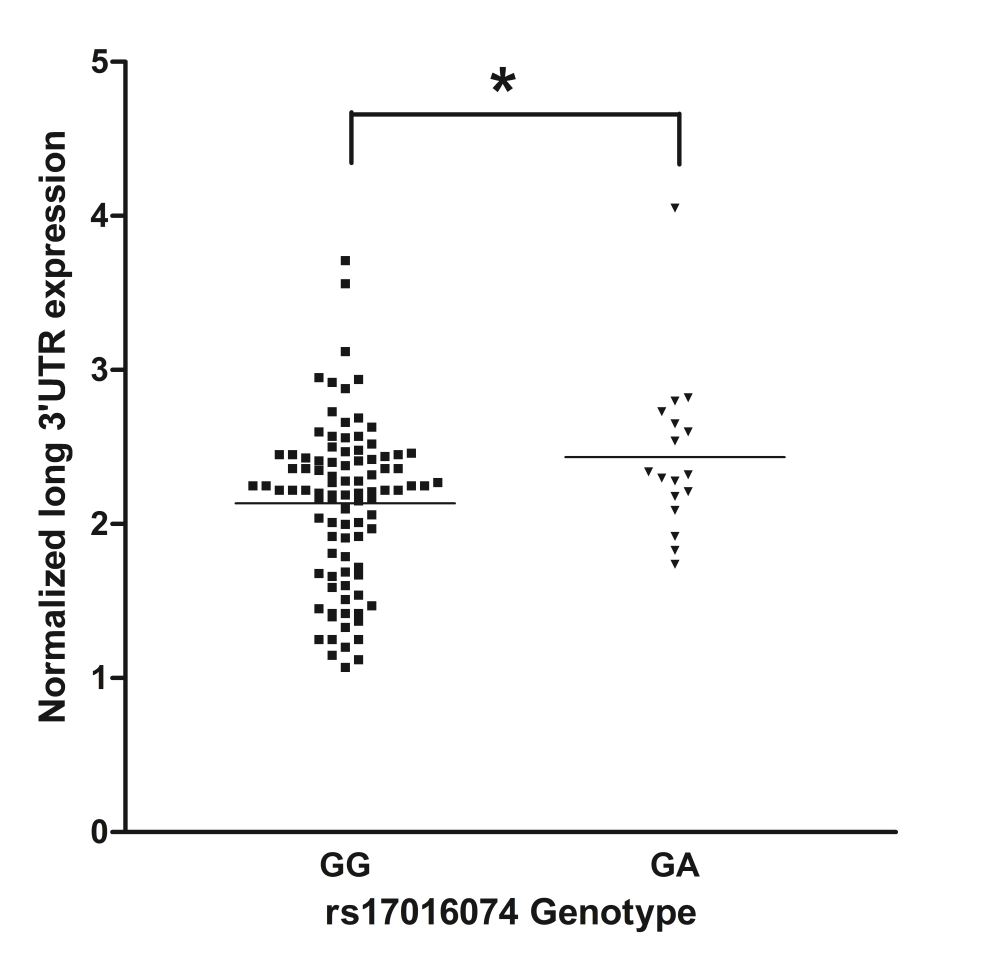


Figure S3. Cycle threshold differences (higher value is lower expression) for full length 3′UTR in human brain grouped by rs17016074 genotype. The data represent the mean (n=2) of qRT-PCR cycle thresholds standardized to overall SNCA mRNA, with PCR primers targeting the 3ʹUTR at the location of rs17016074. There was a significant difference between *GG* and *GA* genotypes for rs17016074 (p=0.03). The minor *A* allele conveys lower mRNA expression (long 3ʹUTR).


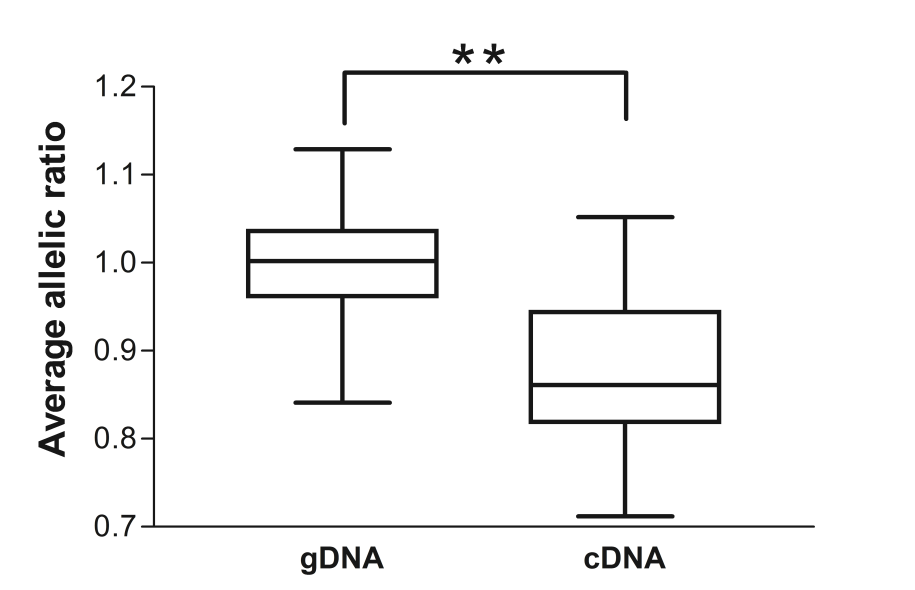


Figure S4. Comparison of allelic ratios in gDNA vs cDNA measured at marker SNP rs356165 for all samples excluding those with large AEI heterozygous for rs1706074 (two-tailed paired t-test p =8.8x10^-6^), demonstrating significant AEI associated with rs356165. The mRNA (cDNA) ratio below 1 indicates higher SNCA mRNA expression supported by the minor *A* allele of rs356165.
